# Gasdermin D inhibition ameliorates neutrophil mediated brain damage in acute ischemic stroke

**DOI:** 10.1038/s41420-023-01349-6

**Published:** 2023-02-08

**Authors:** Ruiyao Hu, Jing Liang, Lan Ding, Wan Zhang, Yuying Wang, Yige Zhang, Ding Zhang, Lulu Pei, Xinjing Liu, Zongping Xia, Yuming Xu, Bo Song

**Affiliations:** 1 Department of Neurology, the First Affiliated Hospital of Zhengzhou University, Zhengzhou, China; 2 NHC Key Laboratory of Prevention and treatment of Cerebrovascular Diseases, Zhengzhou, China

**Keywords:** Stroke, Cell death in the nervous system

## Abstract

Acute ischemic stroke (AIS) induces high level of neutrophils, which correlates inversely with patient survival. Pyroptosis induced by gasdermin D (GSDMD) has been shown to have an important role in the pathophysiology of several inflammatory disorders. The role of GSDMD in the high level of neutrophils after AIS is unknown. Using a middle cerebral artery occlusion (MCAO) mouse model, we identified activation of pyroptosis signal, including expression of caspase-1/11, GSDMD, and interleukin-1β/18 (IL-1β/18), in the brain and spleen at early ischemic injury. Knockout of GSDMD in mice reduced infarct size, improved neurological function, and increased survival after MCAO. GSDMD deficiency decreased the overall degree of inflammation and the proportion of neutrophils in the brain after MCAO. Quantitative studies of neutrophils at several time intervals and organs demonstrated that early inflammatory leucocyte production and supplement (1 day after MCAO) was GSDMD-dependent. A series of bone marrow transplantation experiments, neutrophil depletion experiments, and RNA sequencing results demonstrated that neutrophil specific GSDMD is essential for the production and supply of neutrophil in bone marrow to blood. Moreover, pharmacological suppression of GSDMD decreased pathological abnormalities, infarct volume, and ameliorated neurological function. These results provided a new viewpoint on the immunological modulation of neutrophils after MCAO and suggest that suppression of GSDMD may relieve the neuroinflammatory load, thereby providing a potential treatment strategy for stroke.

**Figure:**
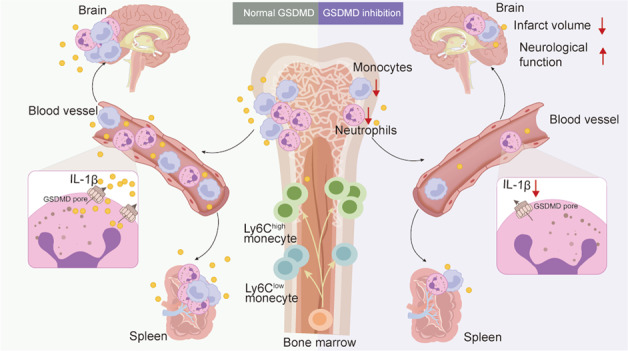
The absence of GSDMD reduces the high level of neutrophils in the brain, the production of neutrophils in bone marrow, and the supply of blood and spleen, while simultaneously the neutrophil-specific GSDMD signal deficiency restrains leukocytosis to improve the pathological outcome of AIS.

The absence of GSDMD reduces the high level of neutrophils in the brain, the production of neutrophils in bone marrow, and the supply of blood and spleen, while simultaneously the neutrophil-specific GSDMD signal deficiency restrains leukocytosis to improve the pathological outcome of AIS.

## Introduction

Acute ischemic stroke (AIS) is a severe neurological disease worldwide and one of the major causes of mortality [1]. Ischemia exacerbates the inflammatory response by activating innate and adaptive immune responses, often resulting in brain damage and lifelong neurological disability [2]. During ischemia–reperfusion injury, messengers such as oxygen radicals activate inflammatory cytokines and upregulate the expression of adhesion molecules on the surfaces of leukocytes and vascular endothelial cells, while inflammatory leukocytes move and adhere to microvascular endothelial cells, resulting in tissue damage caused by inflammatory leukocyte infiltration [3–5]. After ischemic stroke, neutrophils and monocytes are the predominant infiltrating immune cells, and neutrophils infiltration is linked to a poor prognosis [6]. Inflammation involves several regulatory molecules, but the most well-known mediator of inflammation in acute brain damage is the cytokine interleukin-1 (IL-1), which is essential for cytokine production and cell death [7]. Thus, we hypothesize that inflammation-associated programmed cell death plays a role in the pathophysiology of stroke and will become a novel therapeutic target.

In ischemic brain tissue, many types of cell death occur, including apoptosis and necrosis, and pyroptosis is a recently reported inflammation-related form of programmed cell death that requires inflammatory cysteinyl aspartate-specific proteases 1 (caspase-1) and caspase-4/5/11 [8]. After being activated, caspase-1/11 cleaves the inflammatory cytokines IL-1β and IL-18, which causes them to become functional [9]. It has been demonstrated that the presence of pyroptosis markers in brain tissue after stroke, such as NOD-like receptor family pyrin domain containing 3 (NLRP3), caspase-1/11 and gasdermin D (GSDMD), causes an inflammatory response and exacerbates damage to brain tissue and that inhibiting pyroptosis has a neuroprotective effect [10, 11]. GSDMD is thought to be the last executor involved in pyroptosis and the release of inflammatory cytokines after inflammasome activation [12]. The production and release of proinflammatory cytokines such as tumor necrosis factor (TNF), IL-1, IL-6, and IL-18 characterize the inflammatory response to ischemic injury [13]. Consistently, pyroptosis is characterized by cell swelling, membrane rupture and the release of cellular contents, including IL-1β, IL-18, and lactate dehydrogenase (LDH), following the formation of GSDMD pores in the cell membrane, ultimately resulting in a severe inflammatory cascade response [14, 15]. 48 h after traumatic brain injury, the expression of GSDMD and NLRP3 inflammatory vesicles peaks, according to study by Lee et al. [16]. Taken together, these results suggest that GSDMD may be an important regulatory factor for restricting inflammation after stroke. Many investigations have recently shown that GSDMD is broadly expressed in various subpopulations of leukocytes [17]. Monocytes and macrophages serve as sentinels, forming inflammasomes that activate caspase-1 and GSDMD in response to infection [18]. Neutrophils are thought to be the first immune cells to reach the infarct region after cerebral ischemia damage [19]. Two studies showed that in activated neutrophils, GSDMD can be cleaved by neutrophil elastase [20, 21]. Recent findings suggest that GSDMD plays a unique role in neutrophils during inflammasome activation [22]. GSDMD controls neutrophil death and negatively regulates neutrophil-mediated innate immunity, can exert pro- and anti-inflammatory effects and is a unique target for antibacterial and anti-inflammatory therapy [21]. There is a close relationship between neutrophils and pyroptosis in the context of inflammation. However, the function of GSDMD to neutrophils following AIS remains unknown.

In this study, we sought to investigate the effects of GSDMD on neutrophils in a mouse model of middle cerebral artery occlusion (MCAO) and the mechanisms. At the early stage of ischemic injury, the pyroptosis signal was active in both the brain and the spleen. We showed that GSDMD deficiency or pharmacological inhibition diminished neuronal death and infarct volume, and enhance neurological functions in mice. Inflammation and neutrophil levels in the brain, as well as in the blood and bone marrow, were reduced by GSDMD knockout. Neutrophil-specific GSDMD was essential for the production and supply of neutrophils in bone marrow and blood, and also regulated its own signaling pathways, such as chemotaxis. These findings provide a new perspective on the molecular regulation of neutrophil high level after MCAO.

## Results

### The GSDMD-mediated pyroptosis pathway is triggered early in ischemic stroke

**Fig. 1:**
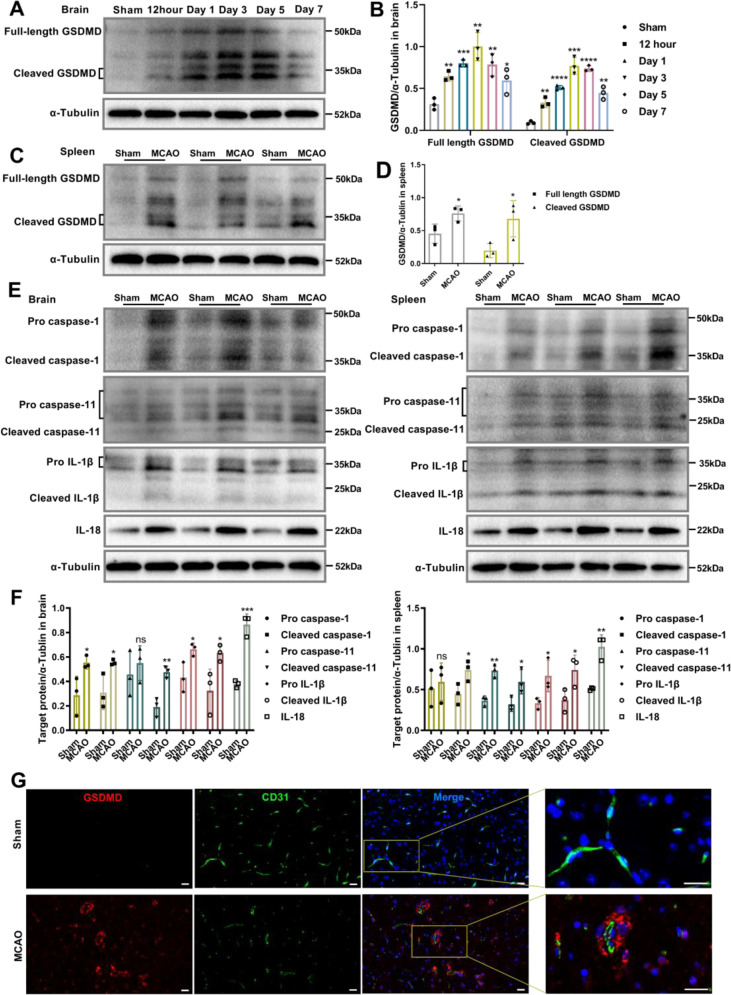
GSDMD is activated in the early phase of ischemic stroke. **A**, **B** Representative immunoblots and quantification of the time course of GSDMD levels in brain of mice after MCAO compared with sham-operated mice (*n* = 3). **C**, **D** Representative immunoblots and quantification of GSDMD levels in spleen of mice 3 days after MCAO compared with sham-operated mice (*n* = 3). **E**, **F** Representative immunoblots and quantification of caspase-1/11 and IL-1β/18 levels in the spleens and brains of mice 3 days after MCAO compared with sham-operated mice (*n* = 3). **G** Representative images of double immunofluorescent staining for GSDMD and CD31. Arrowheads indicate perivascular GSDMD-positive cells. Scale bars, 20 μm. The data are presented as the mean ± SD. Statistical analysis: One-way analysis of variance followed by Bonferroni’s multiple comparison test (**B**) or multiple two-tailed unpaired Student’s *t* test (**D** and **F**). ns not significant, *P < 0.05, **P < 0.01, ***P < 0.001, ****P < 0.0001 *vs* sham-operated mice.

To investigate whether GSDMD is associated with the damage that occurs after AIS, we first analyzed the expression levels of GSDMD in the brains of mice with MCAO at various time points. Immunoblot analysis revealed a significant increase in the expression and cleavage of GSDMD 3 days after MCAO, which persisted until day 7 compared to that in sham-operated mice (Fig. 1A, B). Identical tendencies were seen in the spleen (Supplementary Fig. 1). On day 3 following MCAO, the expression and cleaved levels of GSDMD in the spleen were also elevated compared to those of sham mice (Fig. 1C, D). In addition, the expression and cleavage of caspase-1/11 and IL-1β/18 in the brain and spleen were enhanced 3 days after MCAO (Fig. 1E, F), indicating that GSDMD-mediated pyroptosis is implicated in the pathological process after AIS. Immunofluorescence analysis further showed a high level of GSDMD in the brains of MCAO mice, as well as robust cellular infiltration characterized by a significant number of cells with high GSDMD expression were expressed around the vascular endothelial marker CD31. Notably, positive staining for GSDMD in the brains of control mice was almost undetectable (Fig. 1G). In conclusion, our results suggest that after MCAO, activation of GSDMD-mediated pyroptosis signaling and the release of the proinflammatory cytokine IL-1β/18 occur.

### GSDMD knockout ameliorates brain damage after ischemic stroke

**Fig. 2:**
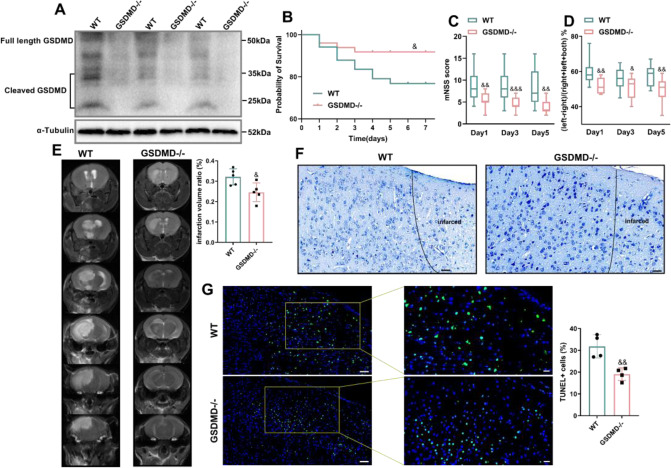
GSDMD deficiency mitigates brain damage caused by MCAO. **A** Western blot analysis of GSDMD protein expression in the brain tissue of GSDMD−/− mice compared to WT mice (*n* = 3). **B** Kaplan–Meier survival curves comparing post-MCAO survival of WT mice (*n* = 35) to that of GSDMD−/− mice (*n* = 50). GSDMD deficiency improved neurological functions, as indicated by the mNSS (*n* = 15) (**C**) and cylinder test (*n* = 13) (**D**). **E** Representative magnetic resonance imaging and quantitative analysis of the infarcted brain on 3 days after stroke in GSDMD−/− mice compared with WT mice (*n* = 5); the infarct area is shown in white. **F** Representative Nissl staining images of the cortex in each group. Scale bars, 50 μm. **G** Representative histology images showing TUNEL staining (*n* = 4). Scale bars, 50 μm. The bar graph shows the percentages of TUNEL-positive cells. The data are presented as the mean ± SD. Statistical analysis: two-tailed unpaired Student’s *t* test (**A**, **E** and **G**), log-rank (Mantel–Cox) test (**B**) or multiple two-tailed unpaired Student’s *t* test (**C** and **D**). ns not significant, &*P* < 0.05, &&*P* < 0.01, &&&*P* < 0.001, &&&&*P* < 0.0001 *vs* WT MCAO-operated mice.

To evaluate the overall function of GSDMD in AIS, we used GSDMD−/− or WT littermate mice that were subjected to MCAO. GSDMD knockout was verified by measuring protein expression (Fig. 2A) and PCR gene detection (Supplementary Fig. 2). We observed that after MCAO, GSDMD−/− mice had a higher survival rate than WT littermate controls on day 3 to day 7 (Fig. 2B). Next, in the same group of mice, we evaluated the mNSS based on spontaneous activity, symmetry in limb movement, forepaw extension, climbing, body proprioception, tactile responses (ranging from 3 to 18; higher scores indicate better prognosis) and sensory function using the cylinder test. GSDMD−/− mice were compared to control mice on days 1–5, and we found that GSDMD−/− animals had improved neurological and sensory functions (Fig. 2C, D). Consistent with these findings, on day 3, the lesion volumes in GSDMD−/− mice were smaller than those in control mice (Fig. 2E). In addition, Nissl staining showed that the cortical neurons adjacent the infarction caused by MCAO were disorganized which had cell edema and necrosis, and that the staining on the Nissl body was shallow. After GSDMD was prevented, cortical neurons were arranged in a more orderly way, and cell swelling and death were lessened (Fig. 2F). Quantification of TUNEL+ cells in the penumbra region further demonstrated that GSDMD deficiency slowed cell death (Fig. 2G). These findings indicate that suppressing GSDMD is effective in protecting mice from ischemic damage by minimizing cell death and promoting functional recovery of the nervous system.

### GSDMD deficiency diminishes neutrophil accumulation in the infarcted brain

**Fig. 3:**
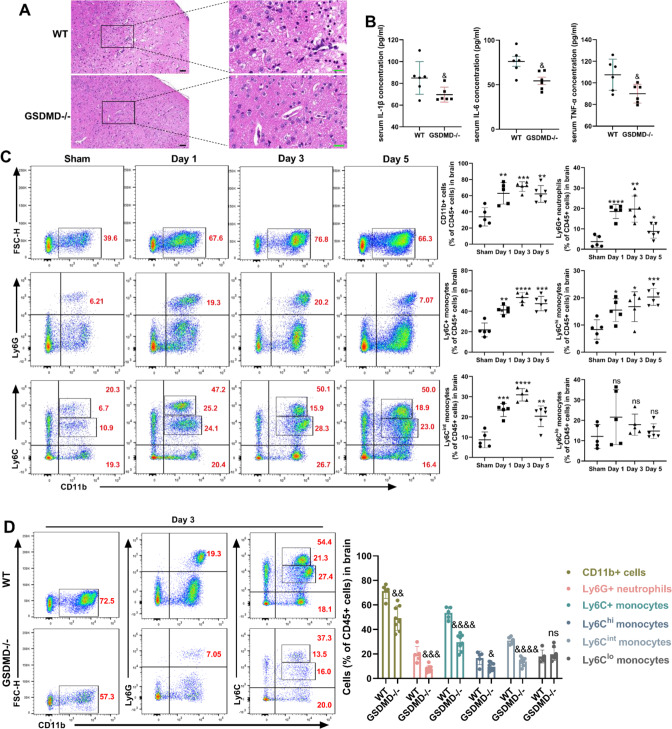
GSDMD is required for the increase in neutrophils in the brain after MCAO in mice. **A** HE-stained coronal section shows the peri-infarct cortical areas at 3 days after stroke in GSDMD−/− mice compared with WT mice. Black bar = 50 μm. Green bar = 20 μm. **B** Quantification of serum IL-1β, IL-6 and TNF-α levels in MCAO mice compared with sham-operated mice (*n* = 6). Flow cytometric gating and quantification of brain leucocytes in (**C**) MCAO-operated mice *vs* sham-operated mice (*n* = 5) and (**D**) GSDMD−/− mice (*n* = 5) vs WT mice (*n* = 8) after MCAO. The numbers inside/next to the gates indicate population frequencies (%). The data are presented as the mean ± SD. Statistical analysis: two-tailed unpaired Student’s *t* test (**B**) or One-way analysis of variance followed by Bonferroni’s multiple comparison test (**C**) or multiple two-tailed unpaired Student’s *t* test (**D**). ns not significant, *P < 0.05, **P < 0.01, ***P < 0.001, ****P < 0.0001 *vs* sham-operated mice. &*P* < 0.05, &&*P* < 0.01, &&&*P* < 0.001, &&&&*P* < 0.001 *vs* WT mice after MCAO.

Numerous studies have shown that the inflammatory response plays a crucial role in determining the prognosis of ischemic stroke. Hematoxylin-eosin (HE) staining revealed that GSDMD−/− mice had less nerve cell loss and intercellular space than wild-type mice, as well as fewer inflammatory leukocytes (Fig. 3A). Therefore, we investigated whether pyroptosis signaling mediated by GSDMD can influence the immunological and inflammatory response in the brains of mice after MCAO. IL-1β is the main cytokine released by GSDMD after pore formation in the neutrophil membrane. IL-6 and TNF-α are the major inflammatory factors released following ischemic stroke. In this study, the absence of GSDMD contributed to a statistically significant drop in IL-1β, IL-6 and TNF-α in the brain 3 days after MCAO (Fig. 3B). Next, we investigated the effect of GSDMD on inflammatory cells in the brain after MCAO. High CD45 expression in the brain was classified as peripheral infiltrating cells, while moderate CD45 expression was classed as microglia. During the 5-day observation period after MCAO, the result revealed that CD11b+ cells increased 1 day after MCAO and reached their peak 3 days later. Among them, neutrophils and monocytes recruitment increased at 1 day in the MCAO group of WT mice and peaked at 3-day (Fig. 3C). Ly6C^hi^ monocytes continued to increase 1–5 days after MCAO, whereas anti-inflammatory Ly6C^lo^ monocytes have no statistical differences during the 5 days after MCAO, consistent with the features of disease-associated cells (Fig. 3C). These data further demonstrate that in MCAO mice models, acute phase management of the inflammatory response is crucial for disease outcome. The levels of neutrophils and monocytes in the brains of GSDMD−/− mice and WT mice were examined 3 days after MCAO. The results demonstrated that the absence of GSDMD considerably decreased the CD11b+ cells, neutrophils and inflammatory Ly6C^hi^ monocytes in brains. Surprisingly, anti-inflammatory Ly6C^lo^ monocytes did not decrease, but a tendency to rise (Fig. 3D). On day 5 after MCAO, GSDMD−/− mice displayed the same outcome, however on day 1 after MCAO (Supplementary Fig. 3A), only neutrophils decreased (Supplementary Fig. 3B). These findings suggest that GSDMD may enhance prognosis by lowering the number of neutrophils in the brain in the early stage of MCAO, which are essential for the inflammatory response in the brain.

### GSDMD deficiency suppresses neutrophil production and supply after MCAO

**Fig. 4:**
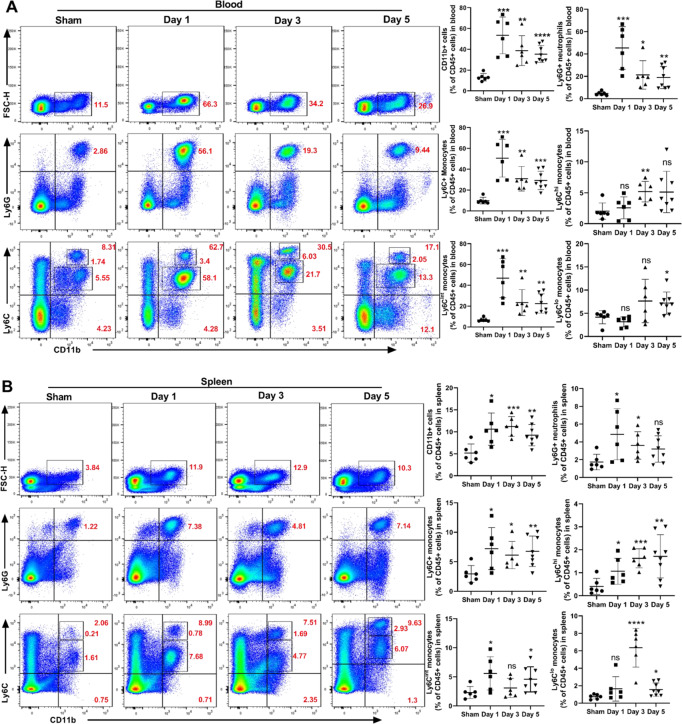

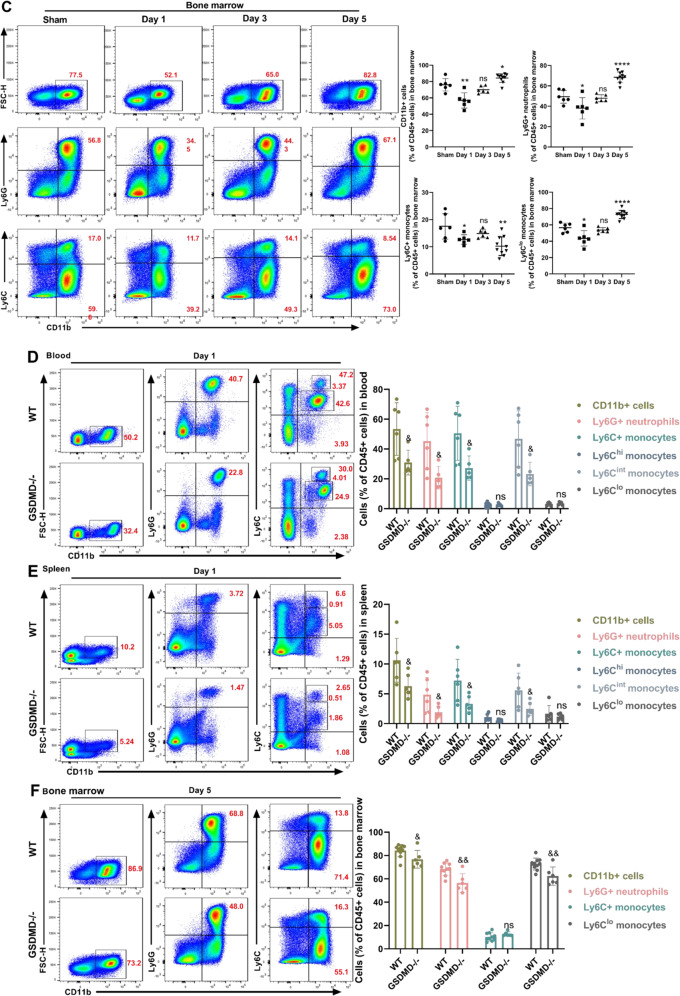
GSDMD deficiency reduces the quantity of neutrophils in the blood, spleen, and bone marrow. **A**–**C** Flow cytometric analysis and quantification of CD11b+Ly6G+ neutrophils and CD11b+Ly6C+ monocytes in the blood (*n* = 6–8) (**A**), spleen (*n* = 6–8) (**B**) or bone marrow (*n* = 6–10) (**C**) of WT mice at different time points (Day 1, Day 3, Day 5) after MCAO or sham surgery. **D**–**F** Flow cytometric analysis and quantification of CD11b+Ly6G+ neutrophils and CD11b+Ly6C+ monocytes in the blood (*n* = 5–6), spleen (*n* = 6) or bone marrow (*n* = 6–10) of WT or GSDMD−/− mice 1 day or 5 days after MCAO. The data are presented as the mean ± SD. Statistical analysis: One-way analysis of variance followed by Bonferroni’s multiple comparison test (**A**–**C**) or multiple two-tailed unpaired Student’s *t* test (**D**–**F**). ns not significant, *P < 0.05, **P < 0.01, ***P < 0.001, ****P < 0.0001 *vs* sham-operated mice. &*P* < 0.05, &&*P* < 0.01, &&&*P* < 0.001, &&&&*P* < 0.0001 *vs* WT mice after MCAO.

Neutrophils are continuously produced in the bone marrow and released into the bloodstream and transported to organs such as the spleen and brain for immune action. To further examine the mechanism by which GSDMD deficiency confers a protective effect on the brain after MCAO and based on recent suggestions that IL-1β, which is a major inflammatory factor released through GSDMD pores in the membrane, play a role in leukocyte production/mobilization [23], we next whether GSDMD knockout would also affect neutrophils in bone marrow and blood. Changes in neutrophils and monocytes (both Ly6C^hi^ and Ly6C^lo^ monocytes) in the blood (Fig. 4A) and spleen (Fig. 4B) during 5 days after MCAO exhibited a similar pattern, and peaks occurred at day 1. This result was accompanied by a significant decrease in the total number of neutrophils and monocytes in the bone marrow 1 day after MCAO, followed by an increase in the number of these cells (Fig. 4C). These suggested that the initial increase in blood neutrophils may have resulted from a mass exodus of neutrophils from the bone marrow, followed by the production of new neutrophils, which was consistent with previous reports [24]. Notably, Ly6C+ monocytes continued to decrease, whereas Ly6C^lo^ anti-inflammatory monocytes increased after a transient decrease in the bone marrow (Fig. 4C). Compared to WT mice, the percentage of neutrophils and monocytes in the blood and spleen of GSDMD−/− mice reduced to various degrees 1 to 5 days after MCAO, with neutrophils possibly being affected more strongly in the early stage (Fig. 4D, E and Supplementary Fig. 4A–D). GSDMD deletion suppressed exclusively Ly6C+ monocytes on day 1 after MCAO (with the lowest percentage of CD11b+ cells). On day 3, the quantity of CD11b+ cells rose, while GSDMD deletion had no effect on leukocyte production. As CD11b+ cell production increased, the proportion of neutrophils in GSDMD−/− mice was considerably reduced (Fig. 4F and Supplementary Fig. 4E, F). These results suggested that suppressing GSDMD expression early after MCAO (day 1) may diminish the overall inflammatory response by lowering bone marrow neutrophil production and blood supply.

### Neutrophil-specific GSDMD contributes to leukocytosis after MCAO

**Fig. 5:**
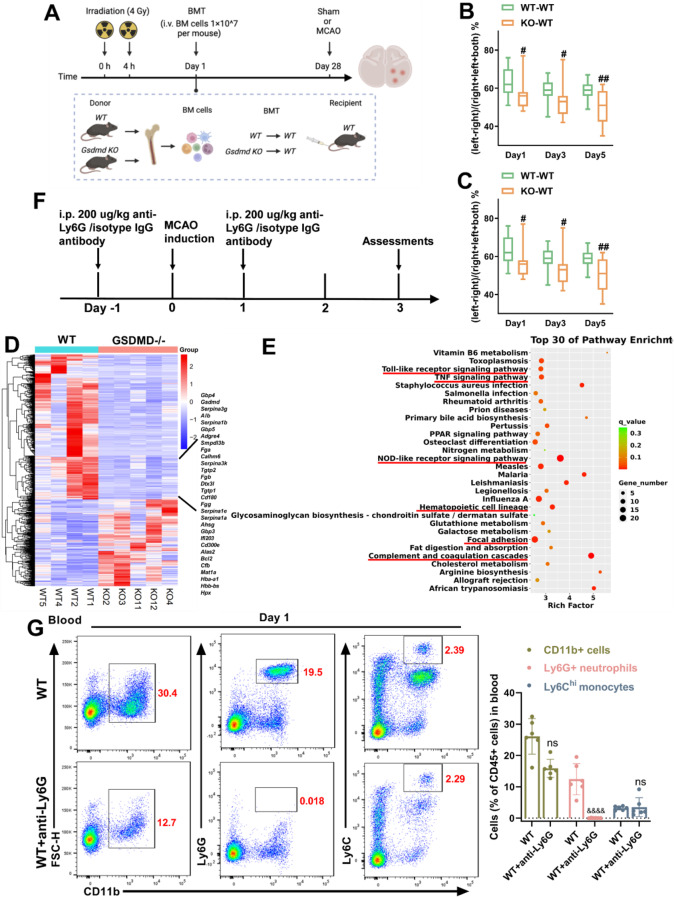

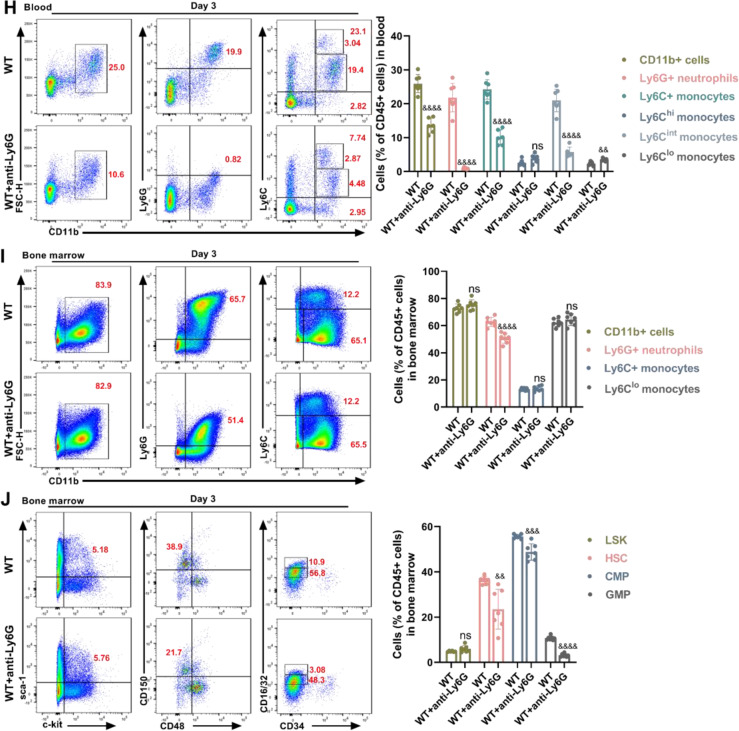
Neutrophil-specific GSDMD contributes to high level of neutrophils after MCAO. **A** Experimental overview: Bone marrow from C57BL/6J WT and GSDMD−/− mice was transplanted into WT recipients, and MCAO was induced after 4 weeks. **B**, **C** Neurological tests were performed to evaluate the motor, sensory, and balance functions of bone marrow chimeric mice on days 1, 3, and 5 after MCAO (*n* = 13–15). **D** Heatmap showing genes identified by RNA-Seq analysis of CD11b+ cells isolated from the blood of WT and GSDMD−/− mice on day 1 after MCAO (*n* = 4–5). **E** GO analysis shows the most significantly enriched signaling pathways in CD11b+ cells sorted from the blood of WT and GSDMD−/− mice on day 1 after MCAO (*n* = 4–5). **F** Flowchart illustrating anti-Ly6G administration and the experimental design. The mice received intraperitoneal (i.p.) injections of anti-Ly6G (200 µg) or an equal volume of isotype IgG 24 h before and after MCAO. **G** Flow cytometric gating of neutrophils and monocytes to validate the successful elimination of neutrophils (*n* = 6). **H**, **I** Flow cytometric analysis of neutrophils and monocytes in the blood (*n* = 7) and bone marrow (*n* = 6–7) between the anti-Ly6G group and the isotype IgG group 3 days after MCAO. **J** Flow cytometric analysis of HSC, CMP and GMP in the bone marrow between the anti-Ly6G group and the isotype IgG group 3 days after MCAO (*n* = 6–7). The data are presented as the mean ± SD. Statistical analysis: multiple two-tailed unpaired Student’s *t* test (**B**, **C**, **F**–**J**). ns not significant, # *P* < 0.05, ## *P* < 0.01, ### *P* < 0.001 *vs* MCAO of WT bone marrow transplantation into WT mice. & *P* < 0.05, && *P* < 0.01, &&& *P* < 0.001, &&&& *P* < 0.0001 *vs* WT mice after MCAO.

Recent studies have shown that Neutrophils move in reverse to the bone marrow, and activated neutrophils produce IL-1β via creating GSDMD pores to increase granulopoiesis [25]. We hypothesized that MCAO-induced neutrophilia requires neutrophil**-**specific GSDMD signaling. First, we constructed bone marrow chimeric mice by lethally irradiating WT mice and then reconstituting the mice with bone marrow from WT or GSDMD−/− mice (Fig. 5A). Through this experimental design, nonhematopoietic cells could activate GSDMD, but myeloid-derived neutrophils could not trigger pyroptosis signaling due to GSDMD deficiency. Consistent with our hypothesis, mice reconstituted with GSDMD-deficient bone marrow performed better on the mNSS and the cylinder test than mice reconstituted with bone marrow from WT donors (Fig. 5B, C). To further investigate how inhibiting GSDMD-induced pyroptosis restricts leukocytosis, RNA sequencing was performed on CD11b+ cells isolated from the blood of WT and GSDMD−/− mice 1 day after MCAO. The heat map displayed the top 30 genes that expression was drastically reduced in neutrophils by GSDMD knockdown. In addition to GSDMD, relevant genes were associated with thrombosis, the interferon signaling pathway, cytokine expression, cell survival, and inflammatory response (Fig. 5D). Kyoto encyclopedia of genes and genomes (KEGG) analysis further showed that toll-like receptor signaling pathway, TNF signaling pathway, hematopoietic cell lineage, NOD-like receptor signaling pathway, focal adhesion and complement and coagulation cascades were top 30 pathways that were downregulated in GSDMD−/− CD11b+ cells (Fig. 5E). To further demonstrate the role of neutrophil-specific GSDMD in the poststroke inflammatory response, depleted reduced neutrophils by intraperitoneally injecting anti-Ly6G antibodies (Fig. 5F). Flow cytometry revealed that anti-Ly6G antibody administration efficiently decreased peripheral blood neutrophils, and did not affect CD45+ cells and monocytes (Fig. 5G). Neutrophils in the blood were dramatically decreased in neutrophil-depleted mice after MCAO compared to isotype control mice (Fig. 5H). Neutrophils in the bone marrow of mice treated with anti-Ly6G antibody reduced 3 days after MCAO, whereas CD11b+ cells and Ly6C+ monocytes did not effect, and the levels of progenitor cells (including hematopoietic stem cell (HSC), common myeloid progenitor (CMP) and granulocyte-macrophage progenitor (GMP)) in bone marrow were decreased (Fig. 5I, J). These findings suggest that leukocytosis after MCAO is dependent on neutrophil-specific GSDMD signaling.

### Pharmacological inhibition of GSDMD diminishes neuropathology, brain inflammation and infarct size after MCAO

**Fig. 6:**
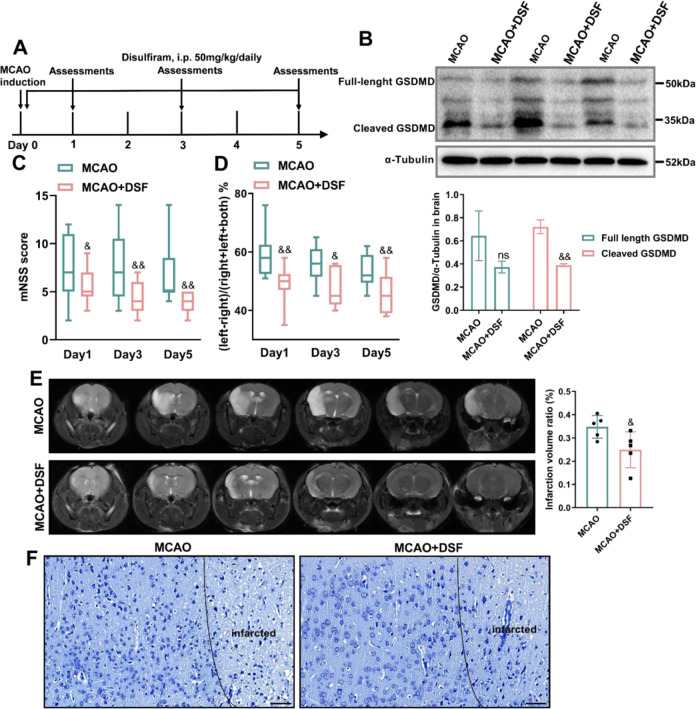
Pharmacological inhibition of GSDMD ameliorates stroke-induced brain deficits. **A** Flowchart showing disulfiram administration and the experimental design. Mice received daily intraperitoneal (i.p.) injections of disulfiram (50 mg/kg) or equivalent amounts of phosphate-buffered saline (PBS) 30 min after MCAO surgery and for 5 consecutive days after MCAO. **B** Western blot analysis of GSDMD protein expression in the brain tissue of disulfiram-treated mice compared with mice in the PBS group (*n* = 3). **C**, **D** Neurobehavioral tests, including the mNSS (*n* = 13–17) and cylinder test (*n* = 13), day 1, day 3 and day 5 after MCAO. **E** Representative magnetic resonance imaging and quantitative analysis of infarcted brains on day 3 after stroke in disulfiram-administered mice compared with PBS group (*n* = 5); the infarct area is shown in white. **F** Representative Nissl staining images of the cortex in each group. Scale bars, 50 μm. The data are presented as the mean ± SD. Statistical analysis: multiple two-tailed unpaired Student’s *t* test (**B**–**D**) or unpaired two-tailed Student’s *t* test (**E**). ns not significant, & *P* < 0.05, && *P* < 0.01 *vs* WT mice after MCAO. DSF disulfiram.

Because disulfiram may pharmacologically reduce pyroptosis [25], it was administered in vivo as a treatment during the early inflammatory response in the MCAO mouse model (Fig. 6A). According to earlier publications, we optimized the dose of disulfiram for in vivo therapy [25]. Protein expression analysis showed that disulfiram significantly reduced the expression and activation of GSDMD (Fig. 6B). In addition to the molecular and neuropathological data demonstrating that pyroptosis in MCAO was inhibited by disulfiram, neurobehavioral examination allowed for repeated evaluations of the therapeutic efficacy. We observed that disulfiram decreased the degree of neurobehavioral impairments in MCAO mice after 5 days of treatment compared to animals that were administered the vehicle (Fig. 6C, D). Consistently, the infarct volume in the disulfiram group was considerably smaller than that in the vehicle group (Fig. 6E). In the MCAO model, the treatment of disulfiram improved the disorderly distribution of neurons in the cortical region surrounding the infarct regions, as well as the light staining of Nissen bodies and cell edema (Fig. 6F). Based on the mouse MCAO models, inhibiting GSDMD is a possible therapeutic strategy for targeting GSDMD-mediated pyroptosis to treat AIS.

## Discussion

A strong inflammatory response was triggered by ischemia, hypoxia and necrosis in the brain [26]. Neutrophils were the first responders to aseptic injury and were key determinants of the nature of the subsequent inflammatory response [6]. Clinically, neutrophils were associated with major adverse cerebrovascular events in patients with AIS [27], suggesting that reducing the number of neutrophils may have a favorable outcome. Our clinical research further demonstrates the significance of neutrophils. However, the mechanisms that regulate neutrophil elevation remain incompletely understood. In the present study, we provided novel evidence that AIS-induced neutrophil elevation in the brain and blood are associated with higher expression and activation of the pyroptosis effector protein GSDMD. Global knockout of GSDMD or pharmacological intervention with the inhibitor disulfiram attenuated ischemic brain injury, improved neurobehavioral performance, and reduced infarct volume after MCAO. Furthermore, GSDMD deficiency reduced the overall neuroinflammatory response by inhibiting pyroptosis signaling, which results in inhibition of early neutrophil production and supply in bone marrow and blood, as well as reduced neutrophil infiltration in the brain, and also inhibiting the release of pyroptosis-dependent cytokines (e.g., IL-1β) and nonpyroptotic cytokines (e.g., TNF-α and IL-6). Moreover, neutrophil-specific depletion after MCAO reduced the level of bone marrow progenitor cell and inflammatory leukocyte production, reducing the supplement of neutrophils in the blood. GSDMD signaling in neutrophils played a crucial function in neutrophil production and brain migration. Taken together, these findings illustrated the biological basis of GSDMD-targeted therapy and provide evidence that inhibiting GSDMD inhibits neuroinflammation (more neutrophils in the brain) and are a therapeutic strategy for reducing brain ischemic injury in the clinical setting.

GSDMD-mediated pyroptosis is a proinflammatory form of programmed cell death characterized by cell swelling, cell membrane rupture, and the release of cellular contents, including IL-1β, IL-18 and LDH [28]. When stroke occurs, GSDMD is activated, leading to the formation of pores in cell and organelle membranes and triggering cytokine release, oxidative stress, calcium overload, endoplasmic reticulum stress, and mitochondrial dysfunction. These pathophysiological processes are detrimental to different cells in the CNS, including macrophages, neurons and astrocytes [29]. Consistent with this, we found GSDMD inhibition could improve the inflammatory environment of the brain and reduced inflammation-associated neuronal toxicity or death, thereby shrinking brain infarcts and improving the prognosis of stroke. Neutrophils are primarily harmful through inducing nonreflux phenomena, releasing elastic enzymes that can increase tissue damage, or producing reactive oxygen species that contribute to blood-brain barrier disruption to affect outcome, severity, and infarct size. Studies during the last two years have shown that active caspase-1 can promote robust secretion of IL-1β but not IL-18 [30, 31] by mouse neutrophils and that IL-18 is constitutively secreted by human neutrophils even in the absence of inflammasome stimulation [32]. In this study, by measuring the concentrations of the IL-1β in blood 3 days after MCAO, we found that GSDMD significantly reduced the level of IL-1β. These results suggest that GSDMD and neutrophils are closely related.

Consistent with the deleterious consequences of inflammatory leukocyte infiltration, GSDMD−/− mice showed fewer leukocytes and less cortical neuronal edema and disarray. In addition to suppressed high numbers of neutrophils in the brain, we noticed that GSDMD inhibition substantially reduced the neuroinflammatory response by lowering peripheral neutrophil generation and supplement. GSDMD, on the other hand, had a delayed and ambiguous effect on monocytes, which may have contrasting effects on distinct kinds of monocytes, increasing anti-inflammatory monocytes while suppressing pro-inflammatory monocytes. Additional trials are required to investigate this mechanism. Notably, GSDMD is expressed in monocytes/macrophages, endothelial cells, T cells, and microglia. The role of GSDMD in neutrophils is distinct from that in other immune cells (e.g., macrophages), and although GSDMD is required for IL-1β secretion by NLRP3-activated human and mouse neutrophils, activated GSDMD is not located at the cell membrane and does not increase cell membrane permeability. IL-1β secretion by GSDMD depends on the autophagic pathway in neutrophils [22]. The signaling mechanism of GSDMD in post-MCAO neutrophils deserves more research.

Changes in gene expression profiles in human blood after stroke occur mainly in neutrophils [33]. To further examined the molecular mechanisms of neutrophil suppression after stroke in the absence of GSDMD, we performed RNA sequencing of isolated CD11b+ cells in blood from WT and GSDMD−/− mice 1 day after MCAO. According to our results, the absence of GSDMD may participate in the production of neutrophils by modulating hematopoietic cell lineage pathway. Because neutrophils play important and multiple roles in neuroinflammation and repair, complete depletion of neutrophils is not a viable therapeutic option for stroke patients, long-term administration of anti-Ly6G antibodies may be harmful, and key targets need to be identified and precisely regulated to avoid disrupting the overall immune balance of the body. We used bone marrow from GSDMD−/−mice in bone marrow reconstitution experiments. Compared to the sham-operated group, GSDMD-deficient mice with chimeric bone marrow exhibited enhanced motor feeling and other comprehensive functions. Anti-ly6G antibody therapy implies that MCAO-induced proliferation of neutrophils requires activation of neutrophil-specific GSDMD signals; however, more time points and in-depth investigations are required to confirm this. The present study also revealed the downregulation of the toll-like receptor (TLR) signaling pathway, TNF signaling pathway and NOD-like receptor (NLR) signaling pathway in CD11b+ cells from GSDMD−/− mice with stroke. TLR4, TNF and NLR deficiency significantly attenuated ischemic brain injury, brain hemorrhage and hemorrhagic transformation to some extent by decreasing the inflammatory response in the brain. This result further clarified the role of GSDMD in the regulation of the inflammatory response after stroke.

No GSDMD-targeted inhibitors for the treatment of ischemic stroke are used the clinic. Necrosulfonamide (NSA) is a small molecule that improves GSDMD-mediated mortality in a mouse sepsis model by inhibiting the formation of p30-GSDMD pores [34]. Disulfiram [25], a drug that is used to treat alcohol addiction, and BAY 11-7082 [33], a previously identified irreversible inhibitor of the NF-κB pathway, potently inhibited GSDMD pore formation and inflammasome-mediated pyroptosis and IL-1β secretion in liposomes by covalently modifying Cys191/Cys192, which is conserved in GSDMD. Recent studies have shown that the active metabolite of disulfiram inhibits the cleavage of GSDMD in macrophages and protects mice from inflammatory responses and tissue damage caused by NLRP3 inflammasome disorder [35]. In this investigation, the use of disulfiram exhibited a protective effect against stroke-related pathological alterations. Disulfiram inhibited pore formation by GSDMD but not other members of the GSDM family and improved the survival of mice with LPS-induced sepsis [25]. Although these drugs inhibited pyroptosis, it is noteworthy that these GSDMD-targeting drugs showed different effects on GSDMD- and caspase-11-mediated liposome leakage. NSA and Bay 11-7082 partially inhibited liposome leakage by inhibiting caspase-11 but were still much less potent than disulfiram [33]. Taken together, these data suggest that disulfiram may be the only direct and effective inhibitor of GSDMD presently available. The current study used disulfiram to effectively inhibit MCAO-activated GSDMD, which was consistent with previous studies. Furthermore, effective pharmacological inhibition was shown to improve neuropathology and motor sensory function after MCAO. Disulfiram may be an ideal molecular drug for therapeutic intervention by specifically inhibiting GSDMD and ideal liposome leakage performance.

In summary, our findings revealed that bone marrow-derived and GSDMD-dependent neutrophil generation and mobilization contribute to detrimental immunopathology after AIS. Inhibiting GSDMD is sufficient to suppress the overall neuroinflammatory response, shedding new light on the inhibition of excessive early (1–5 days) neutrophil mobilization and brain neutrophilia and informing future anti-inflammatory interventions in patients with AIS.

## Materials and methods

### Mice and treatments

Adult wild-type (WT) C57BL/6J mice were purchased from SPF Biotechnology Co. (Beijing, China). C57BL/6J GSDMD−/− (global knockout) mice were purchased from Shanghai Model Organisms Center, Inc (Shanghai, China). Knockout of the GSDMD gene was validated by genotyping and immunoblotting. All animal surgeries were performed on male mice aged 8–12 weeks and weighing 22–25 g. Each group of mice was allocated at random. All animals were housed in a pathogen-free environment in the Zhengzhou University Animal Facility and were given ad libitum access to food and water, housed with nesting material and shelters, and kept in rooms with temperature control and light/dark cycles. All animal experiments were approved by the Zhengzhou University Animal Research Committee.

To deplete neutrophils in mice, isotype IgG and anti-Ly6G antibodies (127601, BioLegend, California, USA) were injected intraperitoneally at a dose of 200 µg per mouse, as described previously [36, 37]. The relevant mice were subjected to transient cerebral ischemia surgery 24 h later.

For disulfiram administration, 50 mg/kg body weight disulfiram (T819460, Macklin, Shanghai, China) dissolved in corn oil was injected intraperitoneally 30 min after MCAO surgery and for 5 consecutive days after the surgery. Mice in the corresponding sham operated group were injected with the same amount of vehicle.

### Ischemic stroke model

Transient cerebral ischemia due to MCAO was induced by using the intraluminal filament technique, as previously described [38]. A silicone-coated nylon monofilament (L1800, Jialing Biotechnology, Guangzhou, China) was inserted into the middle cerebral artery via the right internal carotid artery and left in situ for 90 min. In sham-operated mice, the suture was inserted but not advanced. Mice from the same litters were randomized to receive MCAO or sham surgery. The operation time per animal did not exceed 15 min. During the MCAO procedure, the mice were kept on a heated pad at 40 °C to maintain a core temperature of 36.5° ± 5 °C. The incidence of injury was >70%, and no bleeding was associated with reperfusion. The 10–20% of animals that did not die or develop focal cerebral ischemia were eliminated.

### Neurobehavioral evaluation

Neurofunctional assessments were performed in strict accordance with double blinding principles. All mice were pretrained for 14 days prior to surgery until they reached a steady state of performance. Mice with neurological deficits were evaluated using the modified neurological severity score (mNSS), which comprehensively evaluates motor, sensory, reflex and balance through a series of tests. Neurological symptoms were scored on day 0, day 1, day 3 and day 5 after ischemia. The scores ranged from 0~18, and higher scores indicated more severe neurological dysfunction.

The cylinder test was performed to assess fine sensorimotor function on day 0, day 1, day 3 and day 5 after ischemia. The mice were placed in a transparent cylinder 9 cm in diameter and 15 cm in height. When the mice stood on their hind legs, the number of times the left forelimb, right forelimb and both forelimbs touched the cylinder wall at the same time was recorded for 20 repetitions. The final score was calculated as follows: (left − right)/(right + left + both) × 100%; the higher the score, the more severe the right hemiparesis.

Lesion volumes were assessed 3 days after the induction of ischemia by T2w MRI using a 7.0 T MRS-4717 horizontal animal scanner (MR Solution, Surrey, England), as previously reported [39, 40]. Volumes were calculated using RadiAnt DICOM Viewer software. Infarct volume = (volume of contralateral region − non-infarct volume of ipsilateral region)/(volume of contralateral region × 2) × 100%.

### HE staining

HE staining was performed 3 days after model establishment. Sections were dewaxed and cut into 3 μm-thick sections by conventional methods, stained with hematoxylin for 3–5 min, and then washed with distilled water to remove excess stain. The sections were differentiated with 1% hydrochloric acid alcohol for 1–3 s, placed in 1% ammonia for 20 s, soaked in 0.2% eosin for 4–5 min, rinsed with running water, dehydrated (liter ethanol series), cleared in xylene, and sealed with neutral resin.

### Nissl staining

Nissl staining was performed to assess neuronal survival after MCAO. Coronal cryosections with a thickness of 3 μm were stained with Nissl staining solution (C0117, Beyotime, Shanghai, China) at 37 °C for 5 min on day 3 after modeling. The samples were washed with 95% ethanol for 5 min and dried. The sections were then washed twice in xylene for 5 min each. After being sealed with resin, the cytoplasm was stained blue–purple (Nissl bodies), and the nuclei were stained light blue–purple when viewed under a light microscope. Normal neurons have relatively large cell bodies, are rich in cytoplasm and have a large number of Nissl bodies, with one or two large round nuclei, whereas damaged cells have shrunken cell bodies, condensed nuclei, reduced or no Nissl bodies, dark cytoplasm and many vacuoles.

### TUNEL staining

Neuronal apoptosis was examined 3 days after MCAO using TUNEL staining. TUNEL staining was performed according to the manufacturer’s instructions (11684817910, Roche, CA, USA). Neurons in brain sections or on coverslips were incubated overnight at 4 °C with primary antibodies against NeuN. After the samples were washed with PBST, the TUNEL mixture was added and incubated for 1 h at room temperature, and the samples were washed again and counterstained with DAPI. Positive cells were identified, counted and analyzed by two investigators who were blinded to the groupings.

### Immunofluorescence staining

Mouse brain tissue was embedded in paraffin and sectioned (3 μm thick), and the sections were subjected to immunofluorescence analysis. After being dewaxed, the sections were blocked with PBS containing 0.3% Triton X-100 and 3% BSA at 37 °C for 1 h. Then, the sections were incubated with rabbit anti-GSDMD (1:100, NBP2-33422, NOVUS, Colorado, USA) and goat anti-CD31 (1:40, AF3628, NOVUS, Colorado, USA) primary antibodies overnight at 4 °C. The next day, the sections were rinsed 3 times with PBS and incubated with the appropriate secondary antibodies (1:100, 711-165-152, Jackson; 1:100, 705-545-003, Jackson, Pennsylvania, USA) for 1 h at room temperature. After being rinsed 3 times with PBS, the samples were stained with DAPI solution. Photographs were taken under a fluorescence microscope. Three sections of each brain tissue were randomly stained, and five random fields of view were photographed.

### Tissue preparation and flow cytometry

Mice were sedated under deep anesthesia. Blood samples were obtained from the ophthalmic vein and subjected to red blood cell lysis in RBC lysis buffer (420302, BioLegend, CA, USA). The reaction was stopped with 1× PBS, and the samples were centrifuged at 500 × *g* for 10 min at 4 °C and resuspended in FACS buffer (2% fetal bovine serum (FBS) and 0.01% sodium azide in PBS). The mice were then transcardially perfused with PBS. To collect splenic lymphocytes, spleens were extracted, crushed on the 70 µm cell strainer, and washed in PBS. Bone marrow single-cell suspensions were collected by PBS-rinsing the marrow cavity. Brain tissue was cut into pieces and digested with collagenase D (25 mg/ml, 1108866001, Sigma, Missouri, USA) and DNase I (0.05 mg/ml, 11284932001, Roche, CA, USA) for 45 min at 37 °C with frequent inversions. Afterward, the digested tissue was passed through a 70 μm cell strainer. The cells were then washed, resuspended in 70% Percoll and overlaid with 30% Percoll. The cells were centrifuged at 500 × *g* for 30 min at 4 °C without braking. The interphase was removed, washed, and resuspended in FACS buffer to generate single-cell suspensions.

After the single-cell suspensions were generated, a 10 µl aliquot of each sample was collected, and the total numbers of leukocytes were manually determined using a hemocytometer. Aliquots for cell counting were diluted in trypan blue to distinguish dead from viable leukocytes. Frequencies obtained from flow cytometry were applied to the number of total leukocytes to assess the numbers of leukocyte subsets.

All samples were stained at 4 °C in 100 µl of FACS buffer after the generation of single-cell suspensions. Cells were incubated with anti-mouse CD16/32 (14-0161-82, eBioscience, California, USA) in FACS buffer for 15 min to block Fc receptors. The cells were then resuspended in the desired antibody mixture and stained for 30 min at 4 °C in the dark. After being stained, the cells were washed twice and resuspended in FACS buffer. All samples were evaluated on a BD LSRII flow cytometer (BD Biosciences, New Jersey, USA) and analyzed by FlowJo software (TreeStar, Oregon, USA). The following combinations of antibodies in FACS staining buffer were used: anti-CD45-APC (103112, Biolegend, California, USA), anti-CD11b-FITC (11-0112-82, eBioscience, California, USA), anti-Ly6G-PE/Cyanine7 (127618, BioLegend, California, USA), and anti-Ly6C-PE (12-5932-80, eBioscience, California, USA), hematopoietic lineage antibody cocktail-FITC (22-7770-72, eBioscience, California, USA), anti-CD117(c-kit)-APC (17-1172-82, eBioscience, California, USA), anti-Ly-6A/E (Sca-1)-PE-Cyanine7 (25-5981-81, eBioscience, California, USA), anti-CD48-APC/Cyanine7 (103431, Biolegend, California, USA), anti-CD150 (SLAM)-PE/Cyanine 5 (115911, Biolegend, California, USA), anti-CD34-APC/Cyanine7 (128621, Biolegend, California, USA), anti-CD16/32-PerCP/Cyanine5.5 (101323, Biolegend, California, USA). Leukocytes were identified as CD45+CD11b+ and further classified as Ly6G-positive neutrophils and Ly6C-high/low monocytes. HSC were identified as lin-c-kit+Sca-1+CD48−CD150+, CMPs were identified as lin-c-kit+Sca-1-CD34^int^CD16/32^int^ and GMPs were identified as lin-c-kit+Sca-1-CD34^int^CD16/32^hi^. The data were analyzed using FlowJo software v10.6.1 (BD, New Jersey, USA). For each analysis, cells were first gated on viable (FSC-A vs. SSC-A) and single (FSC-A vs. FSC-H) cells.

### Protein quantification by immunoblotting

Brain or spleen tissue were washed with PBS and lysed in 1× RIPA buffer with protease and phosphatase inhibitors. Protein concentrations were determined by the Bradford assay, and equal amounts of protein were subjected to SDS/PAGE. Then, the samples were transferred onto nitrocellulose membranes. The blots were incubated overnight at 4 °C with the following antibodies: GSDMD (1:1000, ab209845, Abcam, Cambridge, England), Caspase-1 (1:1000, NBP1-45433, NOVUS, Colorado, USA), Caspase-11 (1:1000, ab180673, Abcam, Cambridge, England), IL-1β (1:1000, #31202, CST, Southcarolina, USA), IL-18 (1:1000, ab207323, Abcam, Cambridge, England) and α-tubulin (1:1000, #9099, CST, Southcarolina, USA). The membranes were incubated with appropriate secondary antibodies for 2 h at room temperature. The blots were visualized with SuperSignal West Femto substrate (Pierce) on a ChemiDoc Imaging System (Bio-Rad) and analyzed by ImageJ.

### Enzyme-linked immunosorbent assay

IL-1β (CSB-E08054m, CUSABIO, Wuhan, China), TNF-α (CSB-E04741m, CUSABIO, Wuhan, China) or IL-6 (CSB-E04639m, CUSABIO, Wuhan, China) levels were measured in serum samples obtained from the mice at different time points as indicated in the individual experiments. Briefly, the blood was rested in a microcentrifuge tube for 30 min, centrifuged at 1500 × *g* for 15 min, and stored at −80 °C until analysis.

### Bone marrow transplantation

One week prior to being irradiated, recipient mice were administered water containing neomycin and polymyxin B. Eight-week-old recipient mice underwent lethal bone marrow irradiation by using a small animal X-ray irradiator (Rad Source, Missouri, USA) with two total body irradiations (4 Gy) at 4-h intervals. Bone marrow cells were extracted from femurs, and recipient animals were intravenously administered 1 × 10^7^ bone marrow cells from WT or GSDMD−/− animals by tail vein injection one day after being irradiated. The recipient mice were housed for 8 additional weeks prior to brain ischemic stroke surgery.

### RNA sequencing

For RNA sequencing analysis, CD11b+ cells were isolated from the blood of WT and GSDMD−/− mice 1 day after MCAO using CD11b MicroBeads (130-126-725, Miltenyi, Bergisch Gladbach, Germany). Total RNA was isolated using a RNeasy mini kit (Qiagen, Hilden, Germany). Paired-end libraries were synthesized by using the TruSeq™ RNA Sample Preparation Kit (Illumina, California, USA) according to the TruSeq™ RNA Sample Preparation Guide. Briefly, the poly-A-containing mRNA molecules were purified using poly-T oligo-attached magnetic beads. Library construction and sequencing were performed by Sinotech Genomics Co., Ltd. (Shanghai, China). Purified libraries were quantified by a Qubit 2.0 Fluorometer (Life Technologies, California, USA), validated by an Agilent 2100 bioanalyzer (Agilent Technologies, California, USA) and then sequenced on an Illumina NovaSeq 6000 (Illumina, California, USA). Clean reads were mapped to the mouse genome by HISAT2 (GRCm38.p5). For gene expression analysis, matched reads were calculated and normalized to FPKM. Differential mRNA expression analysis was performed using the R package edgeR. Differentially expressed RNAs with |log2(FC)|value >1 and *q* value <0.05 were considered significantly modulated and were retained for further analysis. We performed a KEGG pathway analysis (http://www.genome.ad.jp/kegg) with the enrich R package.

### Statistical analysis

All data are presented as the mean ± SD. All statistical analyses were performed using GraphPad (Version 9). Statistical analysis was performed by using a two-tailed unpaired Student’s *t* test, log-rank (Mantel–Cox) test or multiple two-tailed unpaired Student’s *t* test. Two-tailed *P* values of <0.05 were considered significant.

## Supplementary information

Supplemental figures

Original full length western blots

Dataset 1

## Data Availability

All data that support the findings of this study are present in this article and its supplementary materials. Additional data related to this paper may be requested from the corresponding author.
