# Supplementary material for: Gasdermin D inhibition ameliorates neutrophil mediated brain damage in acute ischemic stroke
Source: Cell Death Discov. 2023 Feb 8;9:50. doi: 10.1038/s41420-023-01349-6 (PMC9908898; doi:10.1038/s41420-023-01349-6)
Supplement: Supplementary file 1 — Supplemental figures [file 41420_2023_1349_MOESM1_ESM.pdf]

Correspondence and requests for materials should be addressed to Ruiyao Hu or Bo Song.

Supplement figure

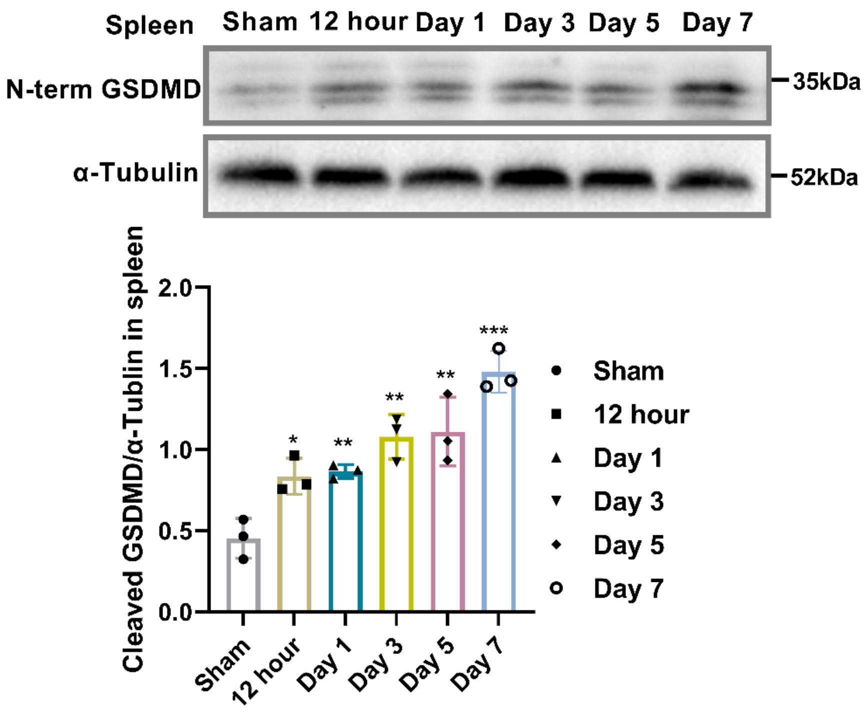

Supplement figure 1. Representative immunoblots and quantification of the time course of GSDMD levels in brain of mice after MCAO compared with sham-operated mice (n = 3). The data are presented as the mean ± SD. Statistical analysis: One-way analysis of variance followed by Bonferroni's multiple comparison test.

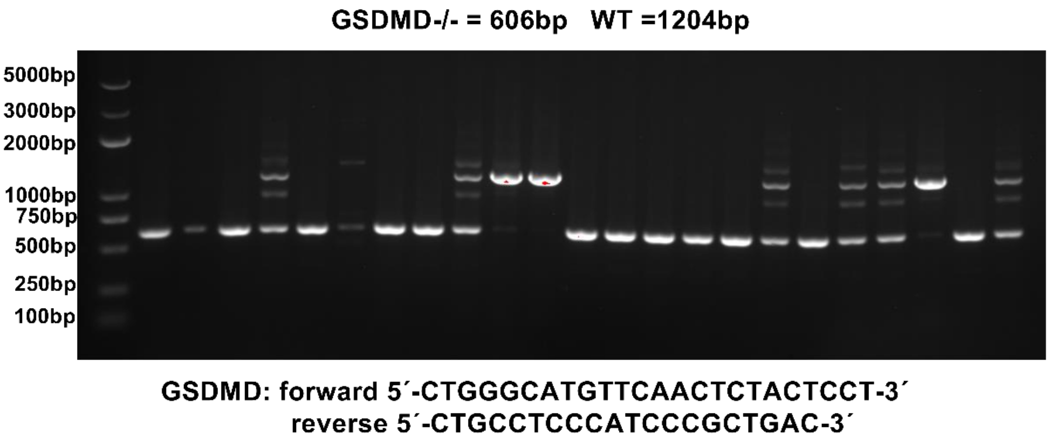

Supplement figure 2. Representative PCR image was used to identify WT and GSDMD-/- mice.

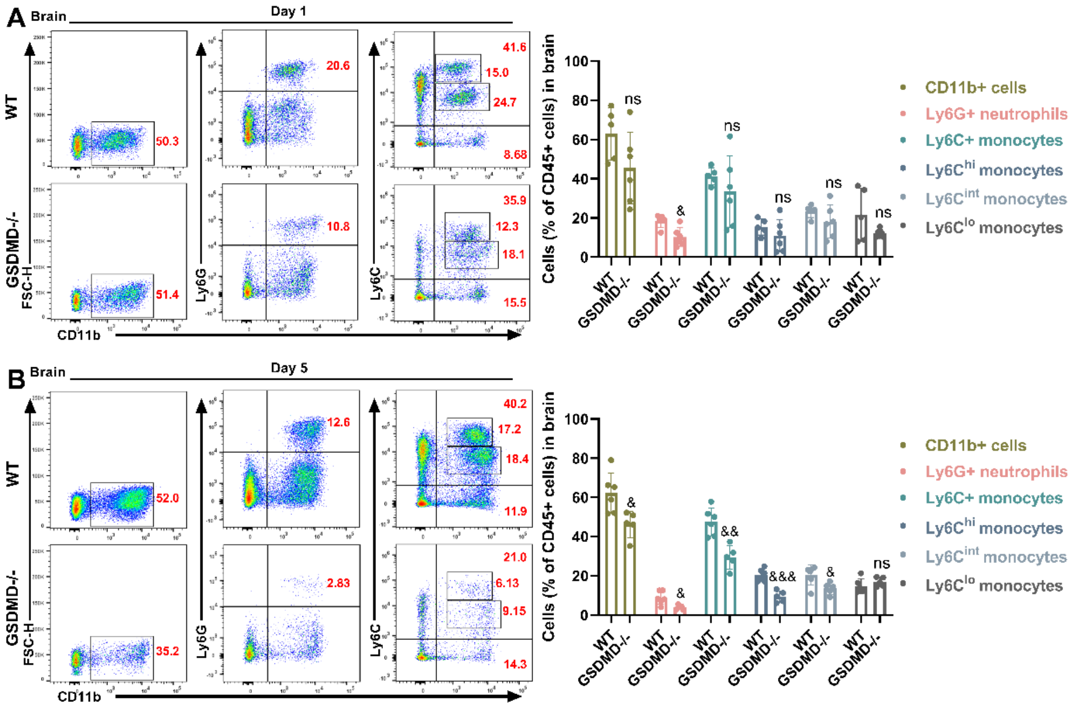

Supplement figure 3. (A-B) Flow cytometric gating and quantification of brain leucocytes in GSDMD-/- mice vs WT mice (n = 5) after MCAO. The numbers inside/next to the gates indicate population frequencies (%). The data are presented as the mean ± SD. Statistical analysis: multiple two-tailed unpaired Student's t test. ns, not significant, & P<0.05, && P<0.01, &&& P<0.001 *vs* WT

mice after MCAO.

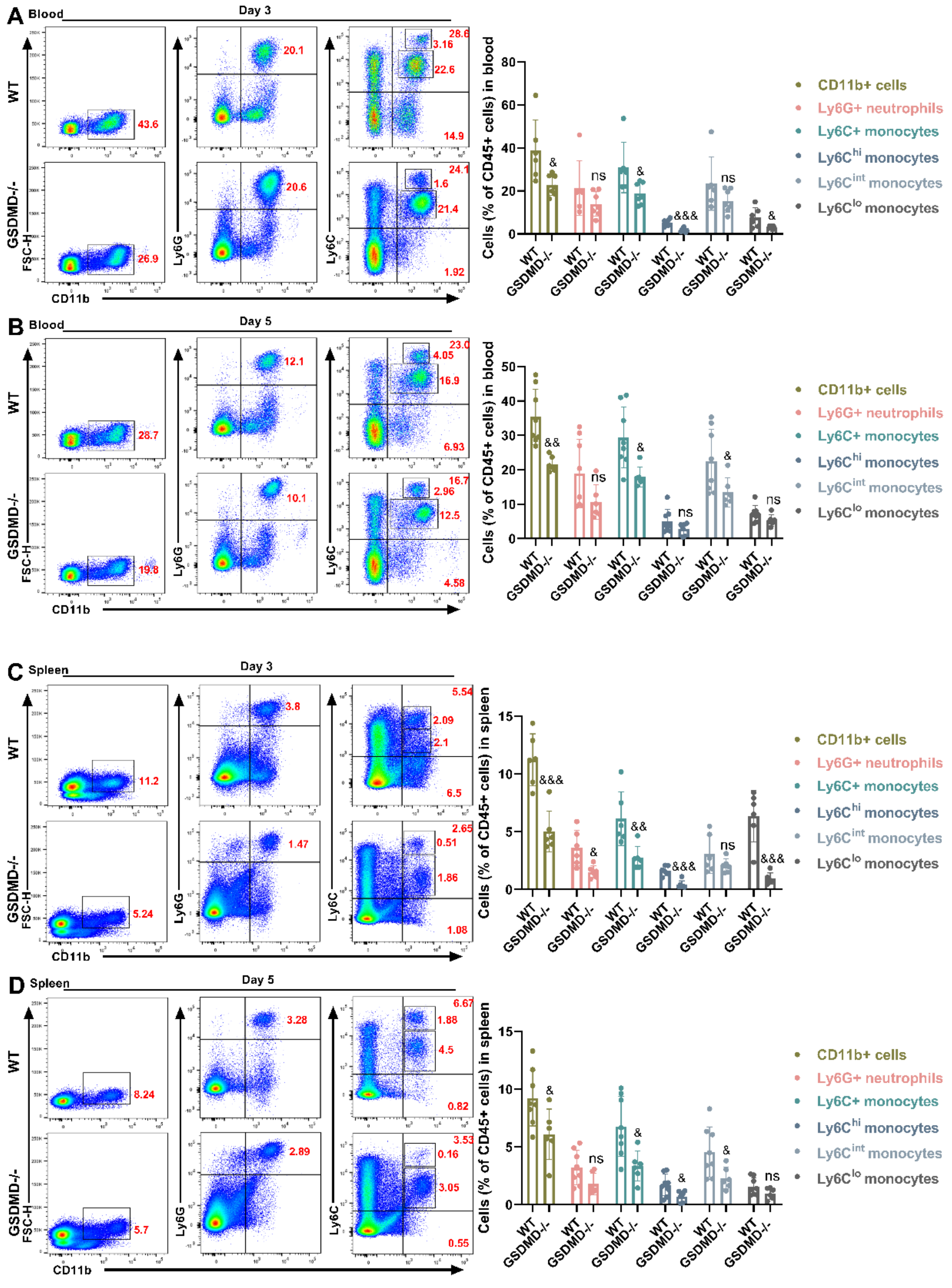

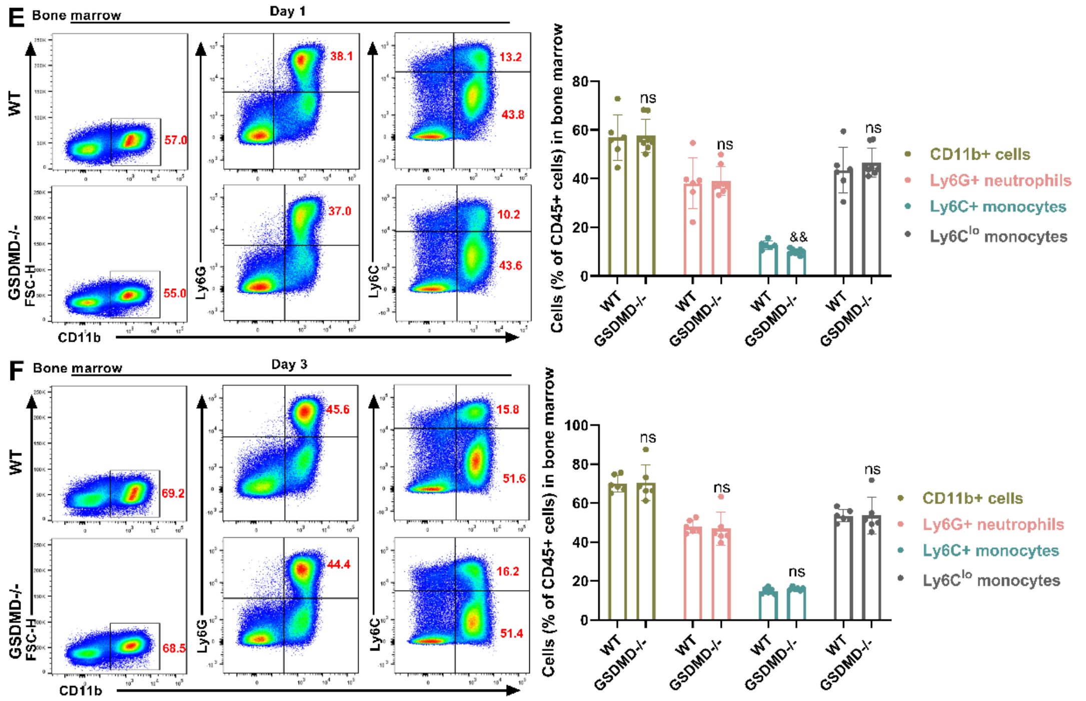

Supplement figure 4. Flow cytometric analysis and quantification of neutrophils and monocytes in the blood (n = 6-8) **(A-B)**, spleen (n = 6-8) **(C-D)** or bone marrow (n = 6-8) **(E-F)** of WT or GSDMD-/- mice 1 day or 5 days after MCAO. The data are presented as the mean ± SD. Statistical analysis: multiple two-tailed unpaired Student's t test. ns, not significant, & P<0.05, && P<0.01, &&& P<0.001 *vs* WT mice after MCAO.
