# Supplementary figures and images for: Gasdermin D inhibition ameliorates neutrophil mediated brain damage in acute ischemic stroke
Source: Cell Death Discov. 2023 Feb 8;9:50. doi: 10.1038/s41420-023-01349-6 (PMC9908898; doi:10.1038/s41420-023-01349-6)

**Original full length western blots**


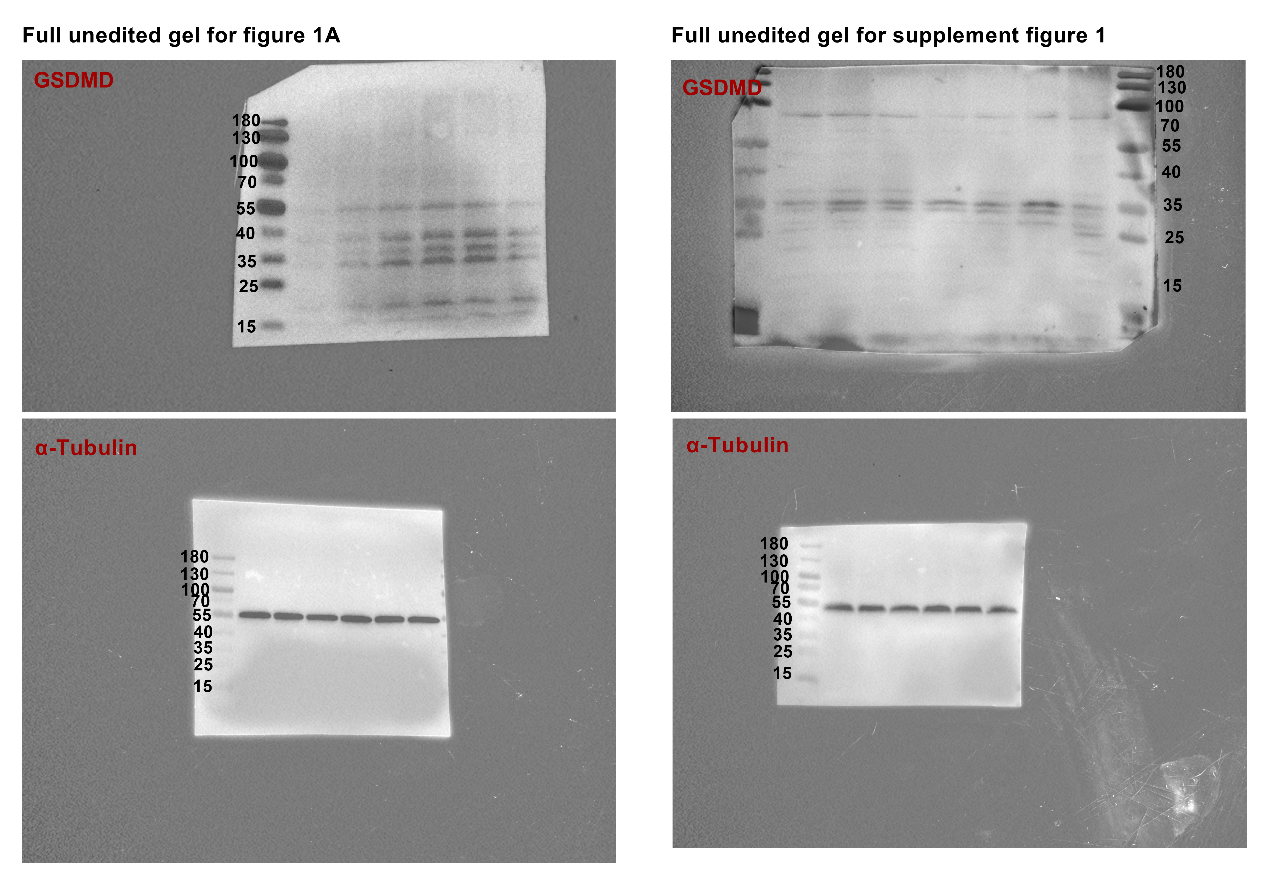


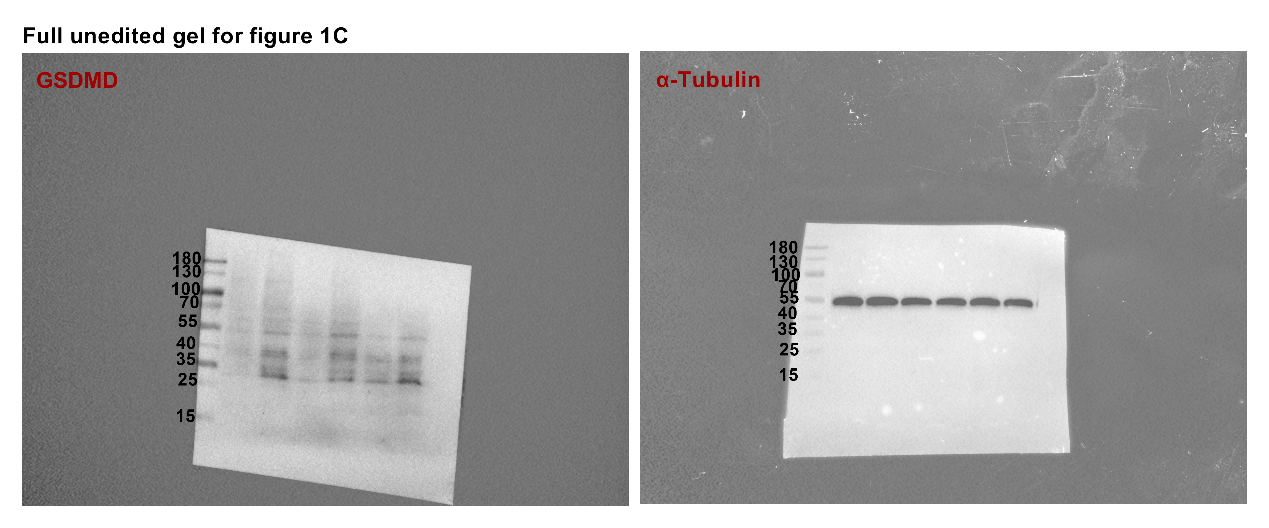


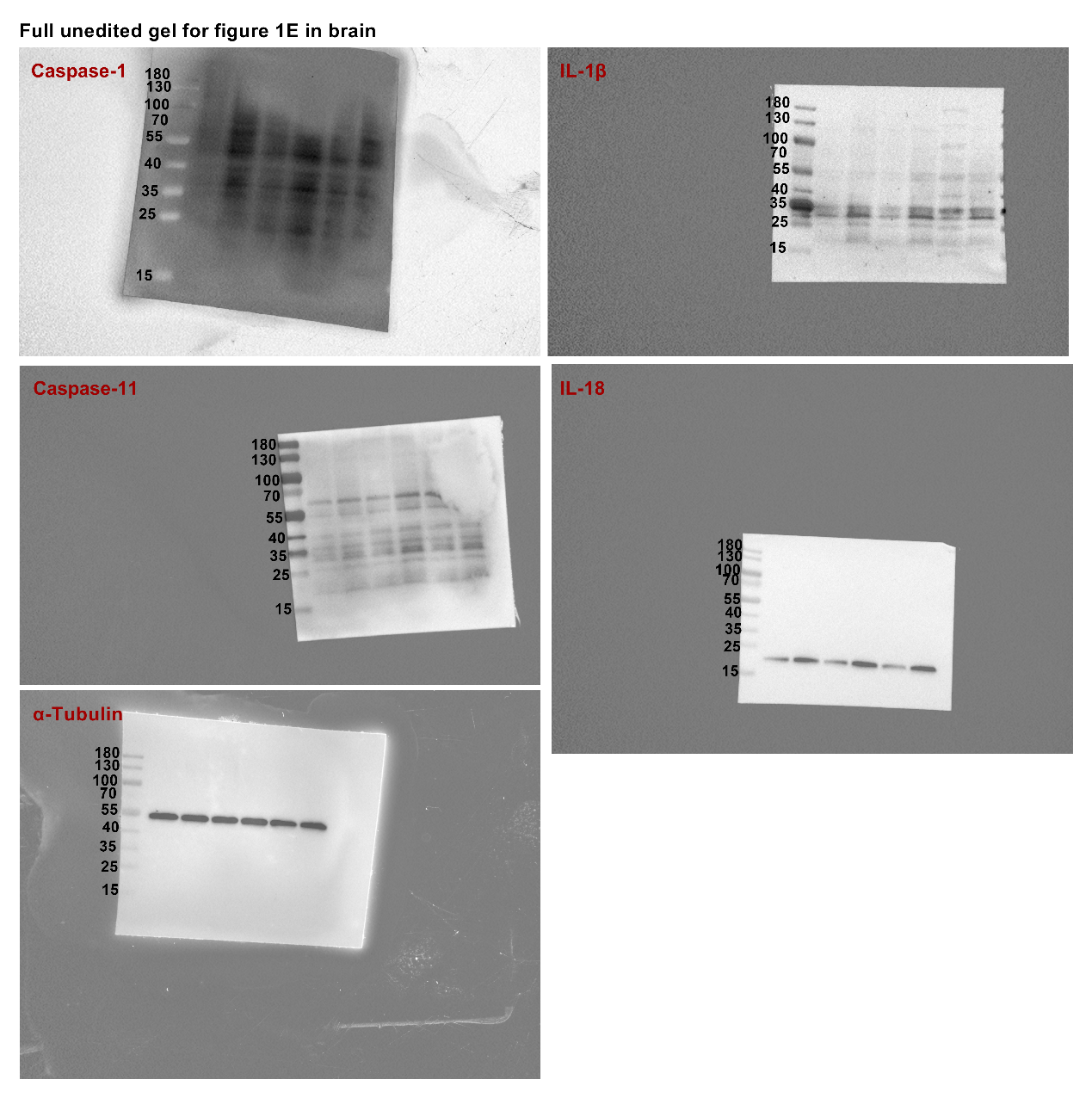


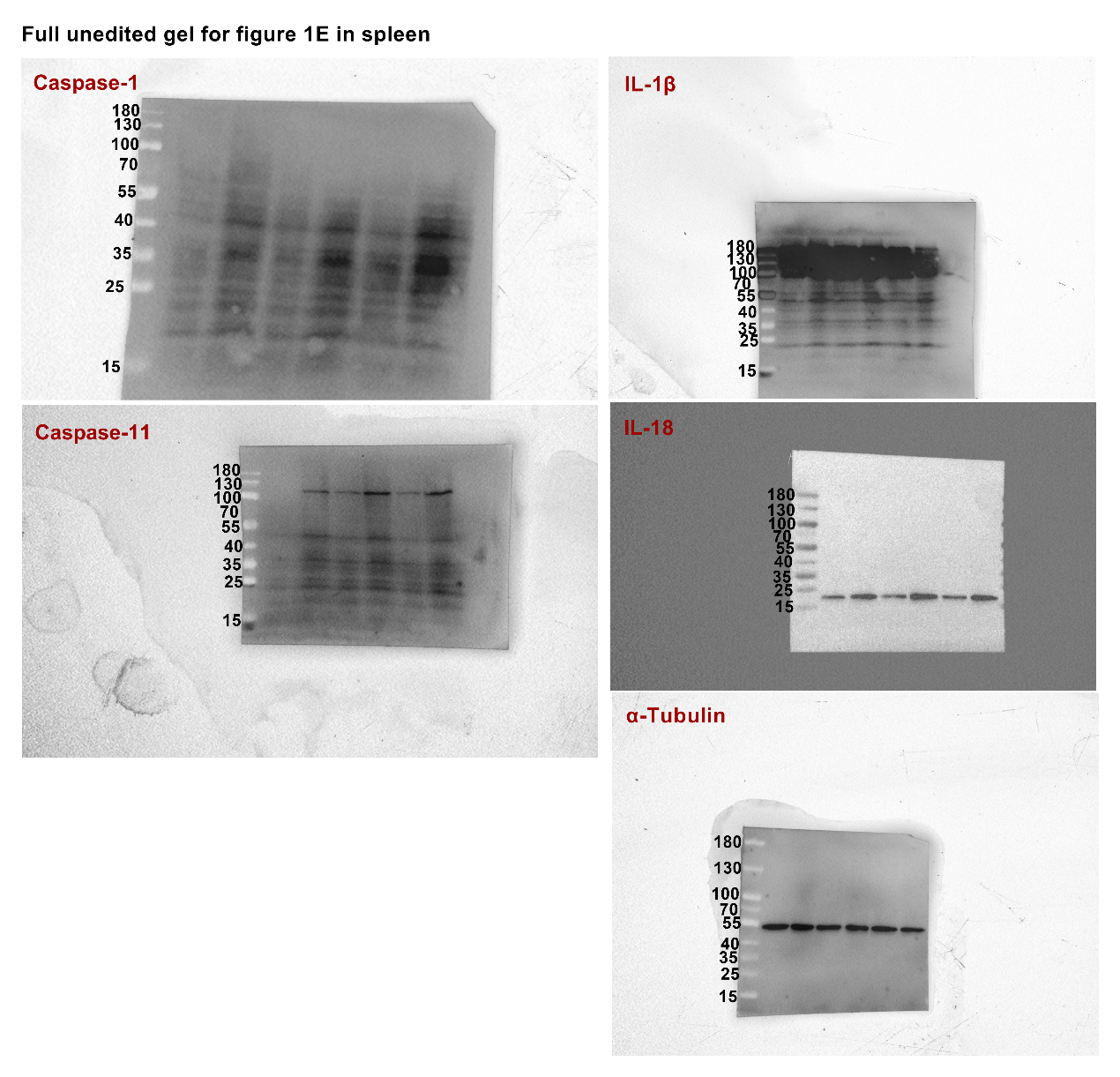


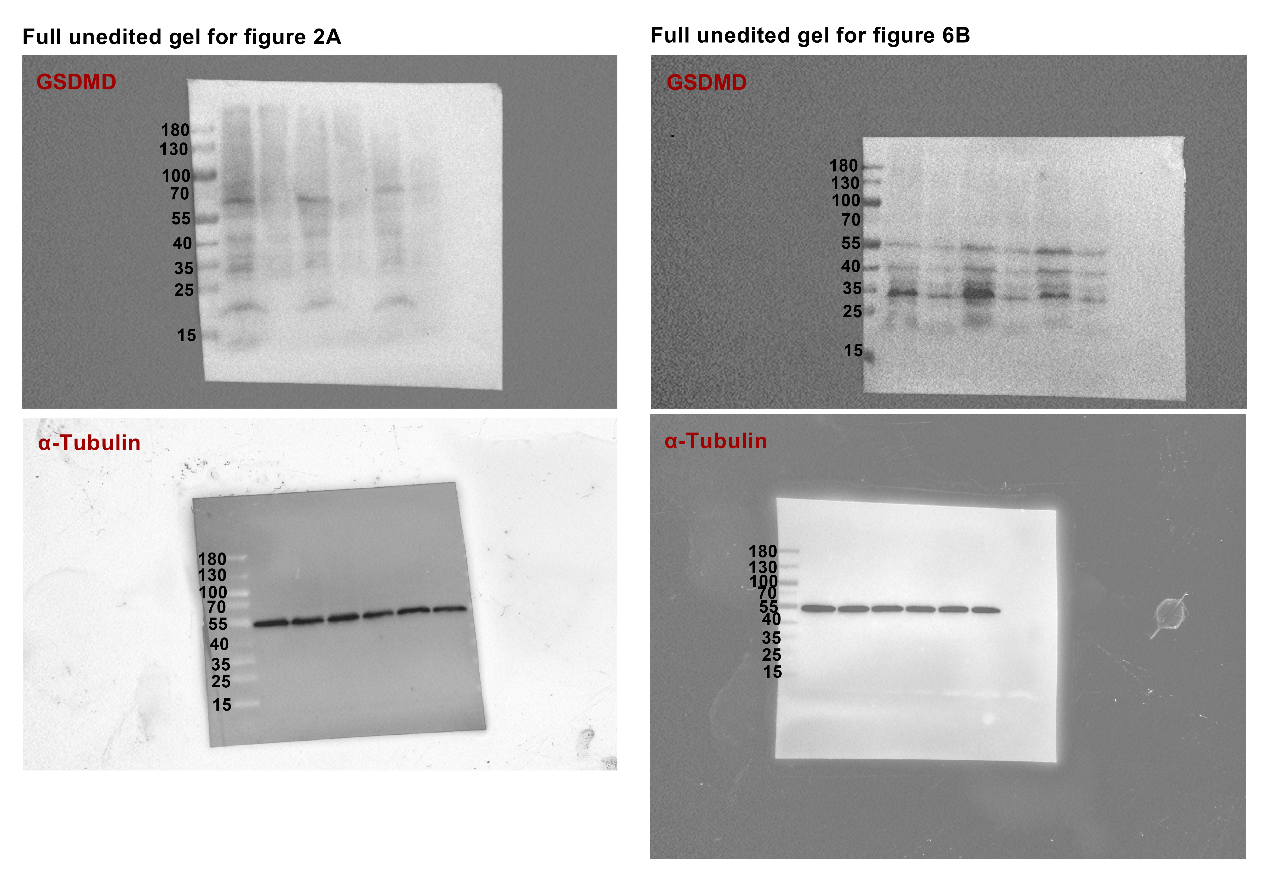

Supplement: Supplementary file 2 — Original full length western blots [file 41420_2023_1349_MOESM2_ESM.docx]
